# Supporting patients to self-monitor their oral anticoagulation therapy: recommendations based on a qualitative study of patients’ experiences

**DOI:** 10.3399/bjgp15X685645

**Published:** 2015-06-29

**Authors:** Alice Tompson, Carl Heneghan, David Fitzmaurice, Stephen Sutton, Sian Harrison, Alison Ward

**Affiliations:** Nuffield Department of Primary Care Health Sciences, University of Oxford, Oxford.; Nuffield Department of Primary Care Health Sciences, University of Oxford, Oxford.; University of Birmingham, Edgbaston.; Institute of Public Health, University of Cambridge, Cambridge.; Nuffield Department of Primary Care Health Sciences, University of Oxford, Oxford.; Nuffield Department of Primary Care Health Sciences, University of Oxford, Oxford.

**Keywords:** anticoagulants, primary care, qualitative research, self-monitoring, self-management

## Abstract

**Background:**

Clinical trials suggest that oral anticoagulation therapy (OAT) self-monitoring is safe and effective, however little is known about the patient experience of this process. There is a lack of understanding about how best to train and support patients embarking on OAT self-monitoring.

**Aim:**

To collect in-depth information about patients’ experiences of OAT self-monitoring outside of clinical trial conditions and to produce a set of recommendations on how best to support such patients.

**Design and setting:**

Semi-structured qualitative interviews with patients who self-monitor and live in England.

**Method:**

In total, 26 of the 267 (9.7%) who participated in the Cohort study of Anticoagulation Self-Monitoring (CASM) and were still self-monitoring after 12 months’ follow-up were interviewed. Topics discussed included experiences of OAT self-monitoring, healthcare support, training, and decision making. Framework analysis was used.

**Results:**

Following initial problems using the monitoring device, interviewees described a mostly positive experience. Although less effort was expended attending monitoring appointments with health professionals, effort was required to conduct self-monitoring tests and to interpret and act on the results. Desire to self-manage was variable, especially when dosing advice systems worked promptly and reliably. Interviewees overcame patchy healthcare system knowledge and support of self-monitoring by educating themselves. Family and friends provided support with learning to use the monitor and managing OAT dosage adjustments.

**Conclusion:**

Better, more-consistent training and health-service support would have alleviated a number of problems encountered by these patients who were self-monitoring. This training and support will become even more important if self-monitoring becomes more accessible to the general population of people on OAT.

## INTRODUCTION

Traditionally, most patients at increased risk of a thromboembolism have been treated with vitamin K antagonists, an oral anticoagulation therapy (OAT) that requires careful monitoring and dose adjustment to ensure the clotting tendency of the blood (assessed using the international normalised ratio [INR]) stays within a specified target therapeutic range.^1^ The consequences of over- or under-coagulation can be grave, with OAT recognised as one of the medicine groups that most frequently causes preventable harm and hospital admission.^2^

In the UK, OAT monitoring services are commissioned locally, with a variety of service designs being utilised; the majority of patients attend monitoring clinics at their general practice or local hospital.^3^ Clinic-based monitoring can be time-consuming and costly, both to the patient and the health service. However, with the development of portable measuring devices that analyse capillary samples it is now possible for patients to self-monitor their own OAT (also called self-testing) and adjust their OAT dosage (self-management).^4^

Clinical trials have indicated that self-monitoring is a safe and effective intervention.^5^ Within these trials, standardised measures have demonstrated statistically significant improvements in treatment-related quality of life;^6^–^11^ however, patient experience of self-monitoring has not been studied in-depth. Furthermore, a significant number of patients who are offered the opportunity to self-monitor decline or discontinue shortly after initiation;^12^ this suggests there are gaps in health professionals’ understanding of how best to train and support patients embarking on OAT self-monitoring.^13^ As a result, this study aimed to undertake interviews with patients who were self-monitoring in order to learn more about their experiences, to identify the barriers and facilitators encountered and to produce a set of recommendations on how best to support such patients.

## METHOD

### Participant recruitment

The Cohort Study of Anticoagulation Self-Monitoring (CASM) was established to investigate whether the positive results achieved in clinical trials of anticoagulation self-monitoring were translated into a ‘real-world’ setting. The full details of the methods and results are published elsewhere.^14^ In brief, 299 people who had decided to self-monitor their OAT were recruited as they purchased a monitor (the CoaguChek^®^ S or XS), either as a first or replacement model, from the major UK distributor (Roche) and followed-up for 12 months. The cohort, therefore, contained members that were new to, or continuing with, self-monitoring. The level of anticoagulation control among this self-selected sample was very good, with few adverse events. Discontinuation rates were lower than anticipated, based on clinical trial data, with 267/296 (90.2%) who began self-monitoring still doing so at 12 months.

**How this fits in**

Clinical trials have indicated that oral anticoagulation therapy (OAT) self-monitoring is safe and effective, but little is known about what patients think of the process and their experiences of it. This study found that, despite sometimes ad-hoc support, self-monitoring had a positive effect on participants’ lives. Better training and robust support systems would have helped address a number of problems that were encountered and will, therefore, be important if OAT self-monitoring becomes more widely available.

In-depth interviews were conducted with a sample of participants once they had completed their 12-month follow up. Among this self-selected sample, the current — sometimes ad hoc — support available appears adequate to enable them to self-monitor successfully. A purposive sampling strategy was used to ensure that a range of backgrounds, opinions, and experiences were studied. Recruitment continued until no new themes emerged. All interviews were conducted by the same researcher.

### Interviews

A topic guide was developed, based on issues that had arisen during the cohort follow up; it included experiences of:

- self-monitoring anticoagulation therapy;
- healthcare support;
- training provided;
- decision making;
- knowledge; and
- quality assurance.

Semi-structured interviews were conducted in participants’ homes or over the telephone and lasted 30–75 minutes. They were digitally recorded, transcribed, and checked to ensure accuracy.

### Analysis

Analysis ran concurrently with data collection to allow refinement of the interview schedule. A framework approach was used to enable both anticipated and novel themes emerging from the transcripts to be identified. NVivo software (version 9) was used to store and organise the data. A coding framework was developed — initial coding was undertaken independently by two researchers to ensure all areas were covered — and refined using the constant comparison method. The One Sheet of Paper analysis method was used to compile all the issues raised for a single code and these were grouped to form themes.^15^

## RESULTS

### Participants

Of 34 CASM participants approached, 26 agreed to be interviewed (older and male participants were less likely to agree) Table 1 summarises their characteristics as collected at the cohort study baseline. Interviewees tended to live in less-deprived neighbourhoods (median Index of Multiple Deprivation Score 2010 of 10.4) and 73% had a professional qualification and/or a degree, generally reflecting the CASM cohort as a whole. Seven interviews were conducted over the telephone due to the interviewees’ distal location from the Oxford-based research team.

**Table 1.** Participants’ characteristics (*n* = 26)

**Characteristic (assessed at cohort baseline)**	***n* (%)^a^**
**Demographics**	
Median age, years (range)	55 (32–78)
Male, *n* (%)	10 (38.5)
Median number of medications, *n* (range)	4 (1–25)

**Occupation, *n* (%)**	
Working full time	8 (30.8)
Working part time	5 (19.2)
Retired	6 (23.1)
Unable to work	4 (15.4)
Home carer	2 (7.7)
Unemployed	1 (3.8)

**Condition requiring OAT (%)**	
Thrombosis	11 (42.3)
Mechanical heart valve	8 (30.8)
Atrial fibrillation	4 (15.4)
Antiphospholipid syndrome	3 (11.5)

**Self-monitoring characteristics**	
Median duration of self-monitoring, months (range)	1.0 (1.0–6.3)
Received in-person self-monitoring training, *n* (%)	19 (73.1)
Under care of an anticoagulation service, *n* (%)	17 (65.4)
Median time in therapeutic range, % (range)	82.7 (37.2–98.6)
Median oral anticoagulation knowledge score (range)^b^	18 (12–20)

**Dose adjustment status, *n* (%)**	
Self-testing	15 (57.7)
Self-managing	8 (30.8)
Collaborative	3 (11.5)

a Unless otherwise specified.

b n = 25, maximum score = 20, assessed at 12 months. OAT = oral anticoagulation therapy.

### Starting to self-monitor

Prior to self-monitoring, the interviewees had few worries about using the monitor; however, the reality, at least initially, did not align with the stress-free procedure described by the manufacturer. One patient said that *‘It must have taken me about 20 goes to actually get it right the first time,*’ (ID336, female, 36 years), while another said *‘It was ridiculous … I was getting really despondent*’ (ID351, female, 73 years).

Problems using the lancet to achieve a sufficiently large blood sample, and applying it correctly to the test strip within the time limit, were encountered. The fact that patients were taking ‘blood thinners’ added to their bewilderment:

*‘I did think … “this is hopeless, I can’t get the blood out, it’s just a waste of time”.*’

(ID342, male, 48 years)

Others felt personally responsible for the problems they were experiencing:

*‘The manual was very clear, written in very idiot-proof language, and then I’ve screwed up.*’

(ID350, male, 32 years)

These feelings were compounded by the use of multiple test strips:

*‘It* [the monitor] *came with a pack of five strips and I think I messed up every strip and I got a bit panicky then.*’

(ID333, female, 63 years)

Over time (sometimes a very short time) some individuals found that using the monitor became less of an ordeal. One male participant explained that *‘It’s awkward at first, but* [*…*] *the more you do it, the more easy it becomes really,*’

(ID338, male, 55 years)

An older female participant said:

*‘I don’t feel apprehensive about it now, I just go ahead and do it.*’

(ID351, female, 73 years)

For others, however, there were hints that, despite use of the device seeming to get easier, it still required some emotional, as well as physical, effort:

*‘I’ve got much more confident now and I don’t feel quite so anxious.*’

(ID332, female, 66 years)

### Training

Although the instruction booklet and DVD supplied by the manufacturer were felt to be helpful, the opportunity for in-person training was welcomed:

*‘I know you can ring the manufacturer and they’re very good, but it’s different, isn’t it when you’re on the other end of the phone? It’s not quite having somebody face to face to see you actually physically doing it.*’

(ID332, female, 66 years)

Training was helpful in overcoming initial problems and extended beyond practical guidance to emotional support:

*‘I had to go back twice because I was getting myself in a bit of a state about it and he* [the anticoagulation clinic nurse] *… very, very, reassuring he was.*’

(ID340, female, 78 years)

The atmosphere in the anticoagulation clinic was less tense than at home. One participant said she thought she:

*‘... was more relaxed because it was in a controlled environment and somebody else was there, an expert was there.*’

(ID333, female, 63 years)

The clinic offered an opportunity to observe practices and to ask questions:

*‘It was a two-way job.* [They would ask] *“What do you think?” Now that was educational.*’

(ID211, male, 76 years)

Some used the clinic practices as a template for their subsequent self-monitoring behaviour:

*‘We’ve got into the routine that* [*…*] *the day that we do* [*…*] *is normally a Friday — only because I used to go to the clinic on the Friday.*’

(ID239, male, 68 years)

Interviewees also educated themselves to supplement the variable levels of support and training available:

*‘I’d researched that* [the monitor] *fully and looked at all the videos and stuff and, by the time the machine came, you know, I just opened it up and I could use it straight away.*’

(ID338, male, 55 years)

### Self-monitoring test results

Generally interviewees trusted the results produced by their monitor; its accuracy was only doubted if the results were outside the participant’s target range. A phase-in period, when the monitor was used alongside clinic testing, was valued by those with limited previous experience of the technology:

*‘Three months or more of running it side by side and then we* [the interviewee and his GP] *decided that the readings were so close that, yeah, it was alright.*’

(ID342, male, 42 years)

A key motivation for self-monitoring was to keep a closer eye on INR levels. However, this was not always the reassuring process anticipated, as the results obtained required interpretation:

*‘Although it* [the monitor] *comes with a lovely DVD and it explains everything and it tells you to do everything, then you think you got this result … and then, “what do I do with it now?”*’

(ID201, female, 39 years)

Knowing how to react, and being able to do so in a timely manner, was valued. This helped mitigate any negative emotional feelings associated with the result:

‘[It was] *fine, because I had everything around me to know what to do immediately … I mean obviously it wasn’t good that it was so low … but it is quite empowering.*’

(ID281, female, 42 years)

*‘If I can contact the INR clinic I’m not too worried because I know that we can increase the dose and, obviously, sort that out pretty quickly.*’

(ID333, female, 63 years)

Out-of-range results could cause feelings of disappointment:

‘[I felt] *annoyed with myself for, like, not dosing myself correctly, but I think I’ve learnt now, with a bit more experience, that it’s a pretty random drug and there’s no point in beating yourself up over it.*’

(ID350, male, 32 years)

Others recognised that such results generate work:

*‘For months it was up, it was down, it was up, it was down, changing the dosage and, you know, it was frustrating thinking, “Why is it messing?”*’

(ID357, female, 55 years)

### Routines and reminders

Some interviewees talked of developing a self-monitoring routine:

*‘It took a bit of tuning to find the right time and the right place to do it* […] *I started off doing it in the mornings and worked out that that wasn’t the right way forward.*’

(ID350, male, 32 years)

A minority were, however, less organised:

*‘Suddenly I get a guilty conscience, yeah, “Oh God, I’d better do it.”*’

(ID211, male, 76 years)

And reminder systems were frequently used:

*‘I write it in big red writing, I put “BLOOD” on my calendar.*’

(ID340, female, 78 years)

Embedded within the routine was record keeping:

*‘When I take the test, not only do I fill in a reading in my yellow book, but I also write it down in my diary as well and … the date when I need to do my next test.*’

(ID368, male, 69 years)

Most interviewees demonstrated high levels of medication adherence:

*‘I take it like clockwork … I have one of the … daily drug organisers, so I always set that up on a weekly basis.*’

(ID336, female, 45 years)

In addition, family members helped; one participant explained that her *‘husband makes up the week’s drugs into little pots.*’

(ID263, female, 54 years)

Some individuals were also able to transfer skills learned from living with other conditions to self-monitoring their INR:

*‘I test with the diabetic* [blood glucose self-monitoring kit]*, it’s a very similar thing.*’

(ID263, female, 54 years)

A variety of testing frequencies were reported, balancing the reassurance of the result and the work required to achieve it:

*‘Once or twice she’s* [the practice nurse] *said, “Well I suggest you check a bit sooner” because obviously* [laughs] *I like to leave it as long as I can.*’

(ID239, male, 68 years)

Those with stable INR levels were advised to leave up to 12 weeks between tests. This presented challenges in terms of both remembering to do the test and how to use the monitor:

*‘Cos you’re not using it very often you tend to forget and that’s when the panic sets in.*’

(ID379, male, 46 years)

*‘I guess the biggest challenge is always just remembering to do it.*’

(ID327, male, 49 years)

### Lifestyle

OAT has numerous dietary interactions and the same self-discipline and effort that was demonstrated in developing a self-monitoring routine extended to interviewees’ lifestyles. There was, however, a workable balance to be made:

*‘I love vegetables, I love spinach, I love cranberries, broccoli. They say you should try and keep it all even but it’s very difficult.*’

(ID333, female, 63 years)

Disruptions included holidays, work, family, and social commitments. As one interviewee explained:

*‘It is hard to maintain an absolutely steady INR* [*…*] *short of having an absolutely monastic lifestyle …*’

(ID327, male, 49 years).

However, necessary compromises were made when integrating self-monitoring behaviour into daily lives, and those with a less-consistent lifestyle were able to track the consequences more closely:

*‘It hasn’t changed my lifestyle in any way at all but it monitors my lifestyle.*’

(ID211, male, 76 years)

For a younger interviewee this was associated with his pre-illness identity:

*‘Maintaining self-testing makes me feel like I’m a normal person that can do anything I want.*’

(ID350, male, 32 years)

There was some evidence that self-monitoring led to an increase in risky behaviour:

‘[Prior to self-monitoring] *I think you tend to be more careful about what you eat and you’re a bit paranoid really … whereas now, if I just thought, “Oh, I’ll eat so and so”, I can always leave it 2 or 3 days and have a check to see if it has affected it adversely.*’

(ID338, male, 55 years)

However, this was limited and one individual’s response was more typical of the cohort:

*‘It never crossed my mind and I don’t think I would want to do that, to be honest … I’d rather take it* [the OAT] *and be, like, proactive and make sure it’s okay before I do something, not afterwards.*’

(ID346, female, 33 years)

Alcohol intake was discussed by several interviewees:

*‘The big thing about warfarin is you can’t have a drink and, oh, you’ve got to be very consistent, and then the last thing I can be is consistent* [laughs]*.*’

(ID352, male, 55 years)

This man’s lifestyle was discordant with his desire for a stable INR. He explained that he *‘… wanted to be absolutely in range, absolutely, you know. I’d like things to be exact*’ and had begun to adjust his dose to rectify his behaviour and improve his INR control. He believed that:

*‘If I have done something silly I can just do a quick check on it.*’

(ID352, male, 55 years)

These dosage adjustments were against the wishes of the clinic.

### Dose adjustment

A meta-analysis of trial data suggested that the clinical benefits of OAT self-monitoring may be greatest for patients managing their own dose adjustments;^5^ as such, this study investigated attitudes towards this. A major barrier experienced was a lack of healthcare professional support:

*‘I wouldn’t mind doing it myself, but I think that’s taking it out their hands and out of their control, and that’s not what they want.*’

(ID357, female, 55 years)

Most clinics with an established self-monitoring service required patients to sign a contract stating they would not adjust their own OAT:

*‘It sets out just what they expect of me and what I would expect of them as well. It’s very clear.*’

(ID338, male, 55 years)

Self-testing meant interviewees were able to avoid physically attending the clinic, which was often a key motivation for self-monitoring.

When the method for receiving dosing advice worked well, the drive to self-manage was further reduced:

*‘I’d be quite happy to do it* [self-manage] *but then I don’t have any problems dealing with the hospital either because they’re so professional.*’

(ID281, female, 42 years)

In addition, some interviewees gained reassurance from being in regular contact with the clinic:

*‘I do feel confident, a bit, sort of, more secure knowing they* [the clinic staff] *are there. That might be just transferring my, the responsibility somewhere else.*’

(ID330, female, 66 years)

However, several interviewees described difficulties in accessing dosing advice:

*‘You think that you’ve got this wonderful machine, you can get on with your life … but, you know, you’re still ringing up the doctors saying, “Look it’s 4.5 today. What do I do?”*’

(ID339, female, 49 years)

Following a 3-day delay in getting advice, she was provided with a dosing algorithm by her haematologist (her ‘prayer book’) and began to self-manage. In her opinion:

*‘Without guidelines … it doesn’t work. Once I got those guidelines, you know, I’m happy. I’m a lot better,* [a] *more-positive person.*’

(ID339, female, 49 years)

The use of dosing algorithms was limited. Other interviewees reviewed previous dosing decisions or utilised a more ‘trial-and-error’ approach:

*‘There seems to be no rocket science to this, you know; if the reading’s too high you take a bit less, if the reading’s too low you take a bit more.*’

(ID327, male, 49 years)

In some cases, family and friends stepped in to also check doses:

*‘There’s two of us, albeit I do it, but we* [interviewee and wife] *do have a chat about the dosage.*’

(ID239, male, 68 years)

*‘I was very much controlling it and running it myself, they* [father and friend] *were just my sort of … double-checkers.*’

(ID350, male, 32 years)

Interviewees recognised the responsibility of altering their own dose, with one participant commenting that:

*‘It’s like being on insulin … they’re both quite dangerous materials.*’

(ID307, female, 59 years)

However, first-time dose adjustment was sometimes unplanned, unprepared for, and in response to weaknesses in the dosing advice system:

*‘For some reason between 5 o’clock on Friday night and 8 o’clock Monday morning you’re very much on your own with the NHS GP practice.*’

(ID307, female, 59 years)

Other reasons for informally managing dose adjustments included pre-empting advice:

*‘I was sort of doing something I know that they would have asked me to, but then perhaps it’s not right.*’

(ID332, female, 66 years)

In addition, some interviewees preferred a more personal approach than those provided by the computer dosing programs used by some clinics because:

*‘… you have a bit more of a gut feeling about yourself*’

(ID330, female 66 years).

The range of acceptable INR results was often different to, and/or narrower than, the conventionally accepted target therapeutic ranges. For example, participant 357’s target range was between 2.0 and 3.0, however she felt:

*‘If it could just stay at 2.5 that would be fantastic, but it’s 2.7, 2.3, the last test I did it was 2.0.*’

(ID357, female, 55 years)

*‘They* [the clinic staff] *don't like me being above 4, so I like to be 3.6–3.8.*’

(ID352, male, 52 years)

Some felt the therapeutic range was arbitrary and disagreed with taking remedial action only once it had been breached, while two interviewees self-managed by altering their diet rather than their OAT dosage:

*‘I’d rather eat the green vegetables and get my INR naturally down.*’

(ID307, female, 59 years)

*‘I sort of force a lot of greens down myself* [laughs]*, and if it’s* [the INR’s] *the opposite way, I’ll have another drink or I’ll have some alcohol cos I know that will bring it back up again as well. So, I’m doing that really instead of going to the clinic in a way, if I’m honest.*’

(ID332, female, 66 years)

## DISCUSSION

### Summary

Interviewees described OAT self-monitoring as a generally positive experience. Key difficulties at the outset of self-monitoring included using the monitor and gaining health professional agreement and/or ongoing support to do so. Over time, most interviewees successfully established a balance between remembering their self-monitoring routine, reflecting on their INR levels, and being conscious of their lifestyle, without becoming obsessed by it. They embedded personalised self-monitoring behaviour within their daily lives.

### Strengths and limitations

To the authors’ knowledge, this is the first in-depth study of people self-monitoring their OAT that provides insights into their experiences. Patients purchasing their own monitoring devices — and therefore eligible for the CASM study — were younger than typical anticoagulant clinic attendees and well educated. Although they are not representative of all patients that could be offered self-monitoring in the future, their experiences provide an insight into this previously understudied area.

### Comparison with existing literature

In accordance with an earlier study of people with diabetes, interviewees reported that self-monitoring could be hard work.^16^ It was, however, viewed as a more favourable alternative to attending potentially inflexible, overcrowded clinics that were unable to accommodate their work, family, and social commitments

Although those with stable INR levels expended less effort self-monitoring than those with fluctuating INR levels, the latter group reaped greater practical rewards by not having to attend frequent clinic appointments. The description of self-monitoring becoming ‘normal’ and interviewees developing a feel for it echoes the experience of patients self-monitoring their blood pressure.^17^ The range of emotions and actions triggered by self-monitored test results also concurs with those described by patients self-monitoring chronic obstructive pulmonary disease.^18^

A few interviewees reported that managing multiple concomitant conditions made self-monitoring easier by providing transferable skills, unlike a sample of patients with hypertension who felt overwhelmed.^19^ However, self-monitoring still relies on the ability of individuals to effectively communicate with health professionals regarding test results, predominantly when they are aberrant. This presents difficulties, particularly in the out-of-hours setting, when access to advice is limited and patient inertia and anxiety can lead to ill-advised decision making. The need for reassurance regarding self-management dosing decisions has also been observed in diabetes self-management.^20^

The discrepancy between the clinically accepted target range and the patient target range has also been observed in the main CASM study^14^ and in diabetes self-monitoring.^21^ In addition, Meier *et al* previously demonstrated that a narrow range does not necessarily improve control and, therefore, self-monitoring patients could be undergoing additional stress and workload for no clinical benefit.^22^

### Implications for research and practice

Box 1 outlines recommendations for the training and support of patients beginning to self-monitor their OAT based on the study findings. Feelings towards self-management were often ambivalent and, for some, it was interpreted as ‘going it alone’ — a rebranding to ‘shared management’ should perhaps be considered to reflect the typical situation where patients manage on a day-to-day basis with support from health professionals being available if there are any problems. In addition, a phase-in period, in which a patient runs their dosing suggestions past a health professional, could help develop patients’ skills.

Box 1. **Recommendations to improve training and support based on the narratives of interviewees**

**Organisational**
- Ensure staff have experience of using the monitor so in-depth support can be provided
- Implement a robust support system, including out-of-hours care if possible

**Initial training**
- Suggest patients bring a buddy to the initial training — someone who can subsequently provide support and reassurance at home

**Training skills**
- Offer advice on how to achieve a sufficiently large blood drop
- Include skills for effective medication adherence and record keeping
- Encourage reflection on how patients can translate the training into their daily lives
- Acknowledge lifestyle variations and disruption to routine; reflect on ways to mitigate their impact

**Training content**
- Reiterate lifestyle factors that affect oral anticoagulation therapy
- Explain the therapeutic range and the risks of being outside it
- Ensure that the therapeutic range is agreed and clearly documented by both patients and staff
- Provide reassurance about the problems encountered during the initial period
- Acknowledge the limitations of any support system
- Consider a phase-in period for those beginning to self-manage
- Review dosing decisions made by the patient

**Ongoing support**
- Agree a frequency for follow-up, either by appointment, e-mail, or telephone contact

Another area that warrants further research is the use of diet to ‘naturally’ manage their INR. Although some interviewees felt this was preferable to adjusting their medication, the consequences could be unpredictable. How common this practice is, and whether it could — or should — be incorporated into self-monitoring dosing guidelines, could be considered.

In some cases, dosing and testing algorithms developed for clinic use were being applied to self-monitoring patients. Interviewees were being asked to leave up to a 12-week gap in between tests, which led to some anxiety and difficulty operating the monitor. These algorithms were developed to balance patient safety with clinic workload. By not physically attending the clinic, this balance has been altered and, therefore, specific testing frequency advice for self-monitoring patients could be applied.^23^

Among this self-selected sample, the current — sometimes ad hoc — support available appears adequate to enable them to self-monitor successfully. However better, more consistent training, and robust support systems would have alleviated a number of problems encountered. Based on the interviewees’ experiences, a set of recommendations has been developed for improved support. Better support systems will become more important if OAT self-monitoring becomes more widely available.
